# Palmoplantar keratoderma in a middle-aged male

**DOI:** 10.1016/j.jdcr.2025.02.037

**Published:** 2025-03-14

**Authors:** Manisha Siriwardene, Frank Isaacs

**Affiliations:** a Department of Dermatology, St Vincent’s Hospital, Sydney, Australia; b Faculty of Medicine, University of New South Wales, Sydney, Australia; c Dermatology, Phlebology and Fluid Mechanics Laboratory, St Vincent’s Centre for Applied Medical Research, Sydney, Australia

**Keywords:** palmoplantar keratoderma, Vohwinkel syndrome

## Clinical presentation

**Fig 1:**
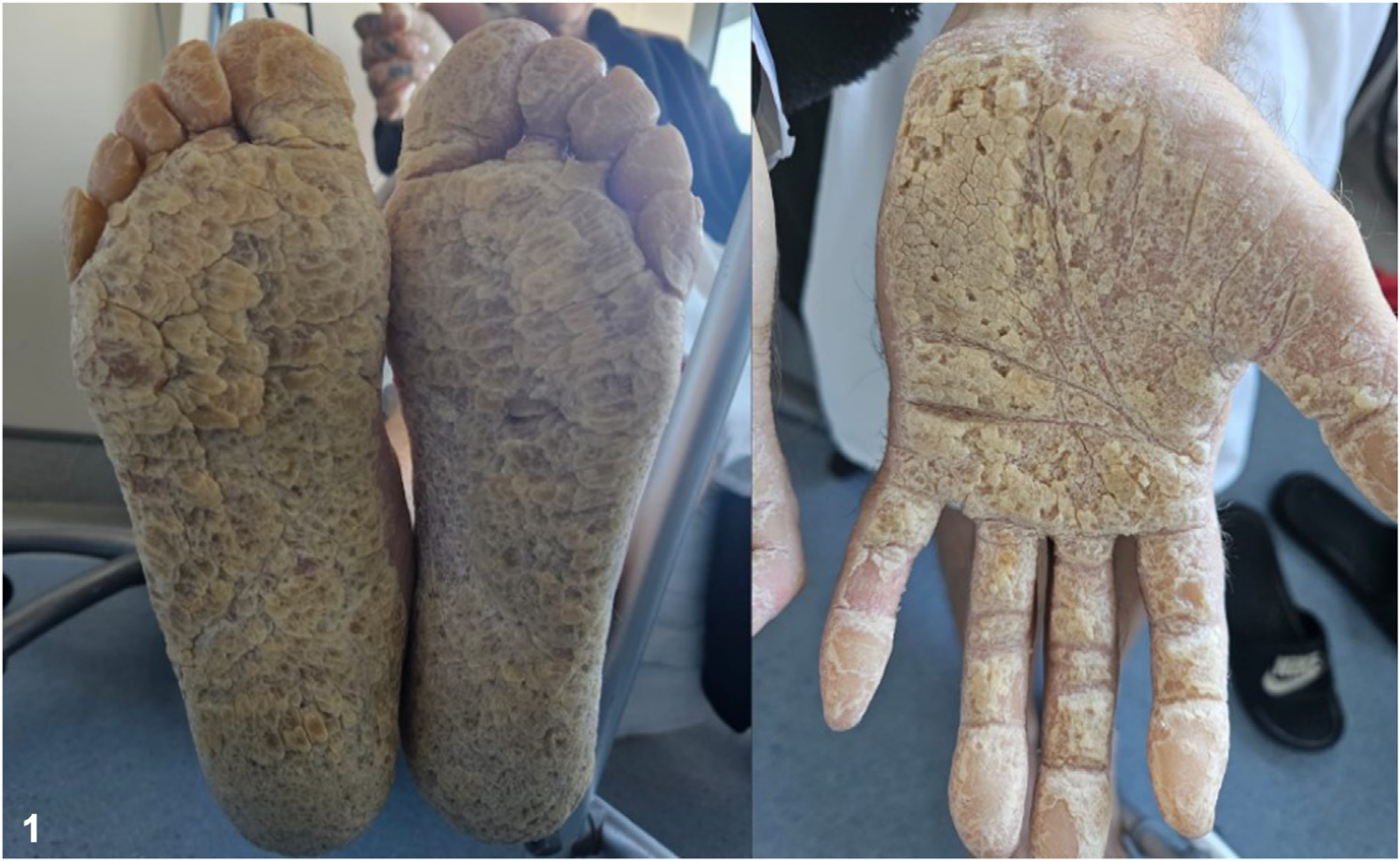
Figure 1 Clinical photograph demonstrating diffuse thickened plaques over the palms and soles of the feet.

A 50-year-old male presented with thick, diffuse plaques over the palms and soles of the feet and a congenital form of sensorineural hearing loss (Fig 1). He was unable to work and required a full-time carer for activities of daily living due to limited dexterity and pain associated with fine movements of the hands and feet. There was a strong family history of palmoplantar keratoderma with a biological father, brother, daughter, 2 nephews and a paternal grandmother also being affected. On examination, there was evidence of honeycomb pattern of hyperkeratosis over the palms and soles of the feet and a “starfish”-shaped hyperkeratosis over the knuckles causing digital constricting bands. There were no ocular defects.

**Question 1: What is the most likely diagnosis?**

A. Olmsted syndrome
B. Vohwinkel syndrome
C. Huriez syndrome
D. Punctate keratoderma
E. Hereditary painful callosities

**Answers:**

A. Olmsted syndrome—Incorrect. Olmsted syndrome is *not* associated with sensorineural hearing loss.
B. Vohwinkel syndrome—Correct. The classical presentation of Vohwinkel syndrome includes a honeycomb pattern of hyperkeratosis over the palms and soles of the feet, sensorineural hearing impairment and a “starfish”-shaped hyperkeratosis typically over the knuckles which may cause digital constricting bands.^1^
C. Huriez syndrome—Incorrect. This condition presents with the triad of congenital sclero-atrophy, hypoplastic nails, and palmoplantar keratoderma.
D. Punctate keratoderma—Incorrect. This condition presents with small round bumps along the palms and soles as opposed to thick, diffuse plaques.
E. Hereditary painful callosities—Incorrect. This condition presents with keratotic lesions over pressure points over the palms and soles; a pattern not demonstrated here.

**Question 2: Which of the following is not a common complication of this syndrome?**

A. Peridontitis
B. Psychologic distress
C. Secondary skin infections
D. Pseudoainhum (digital constricting bands)
E. Sensorineural hearing loss

**Answers:**

A. Peridontitis—Correct. Peridontits is not a complication of Vohwinkel syndrome; however, it may be found in Papillon-Lefevre syndrome.
B. Psychologic distress—Incorrect. Patients with Vohwinkel syndrome often report psychological distress due to increased morbidity and pain associated with their condition.^2^
C. Secondary skin infections—Incorrect. Vohwinkel syndrome may be complicated by secondary skin infections due to breaks in the skin.^2^
D. Pseudoainhum (digital constricting bands)—Incorrect. Hyperkeratosis in Vohwinkel syndrome may cause constricting bands (known as pseudoainhum) which can result in autoamputation of the digits.^3^
E. Sensorineural hearing loss—Incorrect. This is a common complication found in the classical variant of Vohwinkel syndrome.^2^

**Question 3: A mutation in which of genes and correlating protein is associated with this syndrome?**

A. Gap junction protein beta 2 (GJB2 gene); connexin 26 protein^2^
B. Gap junction protein beta 2 (GJB2 gene); protein C
C. TRPV3 gene; connexin 26 protein
D. p53 gene; p53 tumour suppressor protein
E. Fibulin-5 gene; elastin

**Answers:**

A. Gap junction protein beta 2 (GJB2 gene); connexin 26 protein—Correct. A mutation in the GJB2 gene which encodes connexin 26 is associated with Vohwinkel syndrome.^2^^,^^4^
B. Gap junction protein beta 2 (GJB2 gene); protein C—Incorrect. The GJB2 gene does not encode for protein C.
C. TRPV3 gene; connexin 26 protein—Incorrect. The TRPV3 is associated with Olmsted syndrome and does not encode connexin 26.
D. p53 gene; p53 tumour suppressor protein—Incorrect. The p53 gene mutation is not associated with Vohwinkel syndrome.
E. Fibulin-5 gene; elastin—Incorrect. The fibulin -5 gene which encodes for elastin is associated with a form of Cutis Laxa, not Vohwinkel syndrome.

## Conflicts of interest

None disclosed.
